# Insights into Improving Risk and Safety Communication through Environmental Health Literacy

**DOI:** 10.3390/ijerph19095330

**Published:** 2022-04-27

**Authors:** Marti Lindsey, Ben Richmond, Daniel R. Quintanar, Jordan Spradlin, Loren Halili

**Affiliations:** 1 Southwest Environmental Health Sciences Center, College of Pharmacy, University of Arizona, Tucson, AZ 85721, USA; richmond@pharmacy.arizona.edu (B.R.); jordanspradlin@email.arizona.edu (J.S.); lorenhalili@email.arizona.edu (L.H.); 2 City of Tucson, Water Department, Tucson, AZ 85701, USA; dan.quintanar@tucsonaz.gov

**Keywords:** risk communication, environmental health literacy, risk perception, focus groups

## Abstract

Messages and materials developed to communicate risk to the public are often misunderstood because the public misperceives risk, science information is too complex, leading to audience misunderstandings, and an overarching focus on the details of the problem without supplying solutions or actions to keep the public safe. This article describes the creation of a communication model to improve risk communication that includes safety information. The authors describe essential components of Risk and Safety Communication based on features of Environmental Health Literacy (EHL), which informed the creation of a protocol for developing risk communication messages and materials. An online training module was developed to aid communicators in creating information to enable the public to protect themselves, their family, and their community, leading to improved comprehension of how the environment impacts health. These principles were developed in a series of focus groups, identifying how the public perceives risk, how they prefer to receive communication, and how participants respond to materials developed using the principles. Important topics discussed are understanding the literacy levels of the target audience, applying that understanding to developing messages, how risk perception leads to misperceptions and how to address those misperceptions by using plain language when developing focused messages and materials.

## 1. Introduction

This article describes a five-year collaborative partnership between the City of Tucson Water Department–Tucson Water (Utility) and the Community Engagement Core (CEC) of the Southwest Environmental Health Sciences Center (SWEHSC) at the University of Arizona (UArizona). Multiple College of Public Health Interns have participated in this research and provided important insights. The professional development they received has enhanced their academic and professional careers.

The research developed out of studies by Dr. Marti Lindsey and the CEC that had previously been conducted to understand the “Implications of Literacy Related to Comprehension of Environmental Health Materials” [1] and “Knowledge and skills associated with Environmental Health Literacy” [2] and a twenty-year partnership with the Utility developing and disseminating water quality information via the EPA Environmental Monitoring Public Access and Community Tracking (EMPACT) project and other outreach activities to youth and the public in southern Arizona.

Tucson is in the arid environment of the Sonoran Desert and uses groundwater and Colorado River water as the drinking water supply for the region. From the 1940s to the 1970s, industries near the Tucson International Airport (TIA) released trichloroethylene (TCE), 1,4-dioxane, solvents, and other contaminants as by-products of manufacturing. These hazardous wastes went into pits, which seeped into the ground and contaminated an area of the underground water table or aquifer. In 1981, the Utility and the US Environmental Protection Agency (EPA) began collaborating on treating and removing TCE near TIA. In 1993, EPA declared the TIA area a Superfund site/Tucson Airport Remediation Project (TARP) [3]. In 1995, the Unified Community Advisory Board (UCAB) [4,5,6] was formed, comprised of area residents and government agencies interested in preserving groundwater quality and learning more about the process of removing contaminants from the Tucson International Airport Area.

Although there is a long history of water contamination in this area of Tucson, going back to the discovery of trichloroethylene (TCE) in the groundwater water in 1981, 1,4-dioxane in 2001, and Per and Polyfluoroalkyl Substances (PFAS) in 2019 [7,8,9] the Utility has been treating and removing these contaminants from the groundwater and communicating with UCAB about remediation methods for almost 30 years, while providing safe drinking water to this community and all the Utility’s customers. The formation of the UCAB provided the opportunity to establish a dialogue to talk about water quality, treatment, and safe drinking water. Using conventional communication methods has not answered recurring questions and concerns about drinking water quality from the UCAB members, as shown in community responses at meetings of the Unified Community Advisory Board (UCAB) for the Tucson International Airport Area, Tucson, AZ, USA [7], which is attended by the authors of this article.

According to the 2022 World Population by Country [10], Tucson is now one of the poorest big cities in the United States, with a per capita income of just over $20,000. The average household income in Tucson is $58,057, with a poverty rate of 22.45%. Much of the city’s economy is centered on the University of Arizona, which is the city’s second-largest employer, as well as tourism, with over 3.5 million people visiting the city each year. Along with vacationers, there are many winter residents (snowbirds) who come for the mild winters—many own second homes in the area. While the Health Literacy Data Map indicates that most of the population has Above Basic Health Literacy Levels, a further analysis indicates 30–46% of the population has a Below Basic/Basic Health Literacy level [11].

With a 2020 population of 557,718, it is the 2nd largest city in Arizona (after Phoenix) and the 33rd largest city in the United States. Tucson has a population density of 2343 people per square mile. The median age in Tucson is 33.7 years, 32.5 years for males, and 35.2 years for females. According to the most recent census, the racial composition of Tucson is: White: 72.05%, Other race: 10.17%, Two or more races: 5.44%, Black or African American: 5.20%, Native American: 3.68%, Asian: 3.25%, and Native Hawaiian or Pacific Islander: 0.22% [10].

The partners’ experience communicating about water and environmental concerns to general, Tribal, Latinx, and student audiences led to an understanding that the public needs actionable information about how they can keep themselves, their families, and their communities safe from environmental contaminants while at the same time addressing the fear and worry associated with their concerns about water quality. A critical lesson learned was observing outreach and educational material being discarded in the trash at community events such as health fairs.

The ensuing project was developed to demonstrate how risk and safety communication can be aligned to improve the interaction with all audiences, regardless of the level of education, to effectively communicate information on how the audience can remain safe from environmental exposures. The Risk and Safety Communication Model, described in this article, was developed in four phases, each of which included research activities, data analysis, and reflection on the results, which led to the next phase of the research activity.

Phase 1: Eight focus groups

- Conducted to gain an understanding of the water use habits of Tucson residents as well as their thoughts and concerns about water contamination.
- Content analysis of the focus group transcripts using codes developed for this project
- Qualitative analysis of the focus group participants drinking water habits
- Results led to the development of a checklist for materials creation

Phase 2: Pilot test materials created based on the checklist and evaluated

- A common misconception is that turbid or “cloudy” drinking water is contaminated, therefore this topic was chosen to create the pilot test materials
- Multimedia materials included a fact sheet, social media posts, a presentation, and a public service announcement
- These were then evaluated in classroom experiences with public health students
- The results of that materials evaluation lead to the identification of Nine Principles of Risk and Safety Communication

Phase 3: Development of a protocol for message and materials creation

- The protocol was developed to describe the steps needed to create messages and materials that meet the nine principles, which were determined to be the following:
  ○ Identify the audience(s)
  ○ Understand the reason or reasons for the communication
  ○ Use plain language to develop the needed messages–both risk and safety
  ○ Determine the materials that best suit the audience(s)
  ○ Evaluate the understandability of the materials

Phase 4: Creation and testing of an online training module

- The purpose of the training module is to teach public health and utility professionals to use the protocol
- The training module was evaluated with a class of master’s level public health students
- The training module is available in Google Classrooms entitled Risk and Safety Classroom https://classroom.google.com/u/0/h (accessed on 18 April 2022).

The findings of this project describe how the complexity of communicating both risk and safety to all audiences were addressed using the findings regarding plain language [12], environmental health literacy [1,2,13], community partnerships [14], learning about the intended audiences’ level of environmental health literacy and level of trust, can extend the established concepts of risk communication [15].

### 1.1. Background

When a community has concerns about water quality, water utility communicators need to have a proven process in place to provide understandable risk and safety information that will help community members make the best possible decisions and actions for their family’s health, safety, and well-being.

Community members’ knowledge is limited about methods used to treat tap water, risks associated with living in an area with a history of water contamination, and where to find reputable resources on the health effects of exposure to water contaminants. Scientific language used to communicate the risk or safety of a community concern can be difficult for the average citizen to fully comprehend [16].

Additionally, it is crucial to understand why there is distrust between affected communities and their tap water quality [16,17]. Rather than dismissing individuals’ perceived risk as being misinformed, it is important to acknowledge trauma and perceived risk in areas with a history of water contamination, and other environmental health disparities. Because of distrust and risk perception differences, the partnership has found that having accurate and easy-to-understand written and visual information for the public is crucial to effective risk communication practices and to the development of good public relations.

Rather than expecting the public to be knowledgeable about water contamination or other risk issues, it is the communicator who must create messages and materials in a way to be understood by the general public and the affected community.

To develop effective risk and safety information, it is important to know the environmental health literacy (EHL) of the community members of the audience that will receive the communications. However, there is no established measure of EHL. Therefore, the authors have used the health literacy (HL) levels [18] of the community as a surrogate measure. In addition, communicators also need to understand and use best practices of risk communication [15,16,17] when developing messages and materials concerning water quality and any contaminants found by regular testing and other means.

#### 1.1.1. Health Literacy

Health literacy is the ability to obtain, process, and understand the information needed to make health decisions. Health literacy is not only a reflection of an individual’s skills and abilities but also how well health systems provide information and services. Health literacy estimates are based on the 2003 National Assessment of Adult Literacy (NAAL) [18]. This national survey categorized literacy skills into the following four categories: Below Basic, Basic, Intermediate, and Proficient.

“*The results are based on assessment tasks designed specifically to measure the health literacy of adults living in the United States. Health literacy was reported using four performance levels: Below Basic, Basic, Intermediate, and Proficient. The majority of adults (53 percent) had Intermediate health literacy. About 22 percent had Basic and 14 percent had Below Basic health literacy. Relationships between health literacy and background variables (such as educational attainment, age, race/ethnicity, where adults get information about health issues, and health insurance coverage) were also examined and reported. For example, adults with Below Basic or Basic health literacy were less likely than adults with higher health literacy to get information about health issues from written sources (newspapers, magazines, books, brochures, or the Internet) and more likely than adults with higher health literacy to get a lot of information about health issues from radio and television.*”

[18]

Individuals living in communities with lower health literacy levels may be more likely to have problems reading and understanding basic environmental health information, such as a pamphlet about environmental exposure or water quality concerns. Those living in neighborhoods with higher literacy scores may be able to understand basic or water quality health information but could have difficulty with more complex text, such as documents describing medication side effects or specific toxicology information [1,2,5,13]. Knowing the health literacy levels of the audience you intend to communicate with can be helpful in creating meaningful messages and materials for that audience.

A way to view the health literacy levels of your state, county, or census block is to consult the Health Literacy Data Map [11], sponsored by the University of North Carolina, Chapel Hill. This resource provides an interactive, searchable, national map of health literacy estimates for 216,864 census block groups in the United States.

#### 1.1.2. Environmental Health Literacy

In 2014, the National Institute of Environmental Health Sciences (NIEHS) described environmental health literacy (EHL) as

“*an emerging and evolving concept that bridges shared theories from the fields of risk communication, environmental health science, behavioral science, evaluation, communications, public health, and the social sciences. The process of becoming environmentally health literate entails raising scientific literacy, environmental literacy, and numeracy among the general public while increasing awareness of specific exposures and their potential health effects*”.

[13]

Environmental health literacy is an emerging and continually evolving field that combines elements from different disciplines, including health literacy, risk communication, environmental health, communications research, and safety culture [19]. The basics of EHL start when an individual understands the link between environmental exposures and health outcomes. However, the entirety of EHL includes many complex topics. EHL has been an emerging field since the 1950s, and NIEHS had an influential role in producing programs to expand the EHL of populations in the early 1990s [19]. Elevating the EHL of populations gives individuals a chance to take control of their own health as well as to be aware of how their actions may affect the environment around them. Risk communication may be more effective by incorporating an understanding of the knowledge and skills of EHL. The knowledge and skills of being environmentally health literate are described below [2].

An environmentally health literate person knows:

(1) There is a connection between the environment and health
(2) How environmental agents enter the body
(3) Information about harmful environmental agents
(4) Ways to avoid harmful environmental agents but cannot avoid completely
(5) Research takes a long time
(6) Can identify reliable information about the environment

In addition, an environmentally health literate person is able:

(1) Find information explaining how to reduce risks in his/her life
(2) Convey his/her concerns about environmental risks to others
(3) Find information about regional/community environmental hazards/issues
(4) Identify well-known/established hazards in his/her environment
(5) Judge whether an information source is reliable
(6) Find information about hazards in his/her microenvironment, home, or workplace

#### 1.1.3. Risk Communication

Risk is defined as the chance or probability that a person will be harmed or experience an adverse health effect if exposed to a hazard [20]. Risk communication is defined [20] as an exchange of information about the nature, importance, implications, or control of a risk. At the time the study began, the gaps in the literature and guidelines were a lack of consideration of the EHL or demographics of the audience for risk communication, nor did they focus on safety information. The earliest efforts to combine EHL with Risk Communication were two presentations given by author Lindsey at the NIEHS conference, *Communication Research in Environmental Health Sciences: Environmental Health Literacy*, “Building consensus on the skills associated with Environmental Health Literacy”, the keynote, and “Identifying Metrics for Environmental Health Literacy Activities” [21].

Government agencies have increasingly sought improved means for communicating risk information to individual citizens and public groups because they are discouraged by the public’s responses to their efforts to communicate. They think the public’s perceptions of risk and demands for risk reduction are unrealistic. Members of the public often distrust governmental agencies. They feel the government is disinterested in their opinions and concerns and are reluctant to allow community engagement concerning decisions that intimately affect the lives of the public [22]. In 1987, researchers knew that to the public, risk means much more than scientific information. At the time this study began, the belief was that the public pays too little attention to hazards and that experts do not pay absolutely sufficient attention to outrage, so they rank risks differently [23]. At the time this study began, there was little empirical literature concerning how to deal with this difference in perception and practice, with communicators focusing on the scientific information and the public desiring participation and information that is meaningful to them personally.

Current risk communication research has identified disconnections between the stakeholders in risk communication, especially how disconnects occur in the way technical experts and the public view and understand particular risks. Risk communicators have discovered that listening and motivation are keys to understanding [15,17,20].

Misconceptions about risks can result due to differing opinions and muddled information from various sources, some of which are undependable or not credible. Researchers suggest making information available in plain and understandable language to foster effective decision making [12]. Slovic [24], highly regarded on the topic of risk perception, states that perceptions of risk are rooted in social, educational, literacy levels, and cultural factors determined by actual risk information and by information sources a person uses, word of mouth stories, print and television news, and the internet.

Because risk communication is the purposeful exchange of information about risks among stakeholders, government agencies, members of the media, scientists, and community members [25], it is best structured as a collaborative process that takes all stakeholders into consideration to be highly successful. At times too much information is imparted during risk communication, thus complicating the public’s understanding of the situation, which may lead to miscommunication, fear, and lack of trust in the information source [26,27,28].

There are several challenges in communicating risk to the public, as many members of the public want to contribute to the process of decisions being made by government officials regarding risks in their community. Oftentimes, though, there are several factors that can lead to difficulty in communicating risk, including: differing levels of ‘acceptable risk’ on an individual basis, incorporation of differing interpretations of risk communication information available, provision of information that assists in personal decisions as well as informs individuals on active policy [25].

One basis of public health is communicating risk to the public [29]. Utility communicators and public health professionals need to relay vital water quality and health information regarding risk and perceived risk to be effective [17]. Perceptions of risk by the public are determined by actual risk information (i.e., heart disease spanning a family’s ancestry) and by information from external sources, such as word of mouth stories, news articles, television, and the internet. Residents of a community perceive the notion of risk differently; thus, providing accurate and easy-to-understand information for the public is crucial to risk communication practices.

Risk perception is a critical aspect of risk communication, specifically in how the public understands and responds to risk. Sociology and anthropology research states that perception and acceptance of risk have their roots in social and cultural factors [15,17,20]. Individuals within a community have unique perspectives on hazards based open these factors. Unique experiences, upbringings, and cultural involvement all play a role in whether risk is perceived as dangerous for health outcomes [17]. Risk communication and risk management must be structured as a two-way process, or it will fail. Both experts and the public have something of value to contribute, and it is important to respect both sides [29].

## 2. Research Methods

This project is not a systematic exploration of a research question or hypothesis; rather, it is translated from empirical practice to theory and education over several years. Experience told the team that the public was distrustful of their water quality due to a history of trauma. It began with observations that many of the messages and materials disseminated by the partnership were not well received at UCAB meetings and were found in trash cans at health fairs and other community engagement activities.

Thus, this is a case study, which intended to describe, evaluate, and understand aspects of why the communications at the UCAB, health fairs, and other community engagement events were not effective. It grew from materials assessment and focus group questions to the discovery of principles that would make the messages and materials more receptive to the public, especially the focus on how those people could keep themselves and their families safe, to testing pilot materials, developing a protocol to guide communicators in presenting, and finally an online education module for broad dissemination.

The intention of this case study was to better understand the thoughts, feelings, and perceptions that people have about the quality and safety of their drinking water and how they want to receive information, especially what sources and methods people use to receive information about their tap water.

The team, comprised of the authors of the paper, student staff members, and public health student interns, met at least monthly to summarize the findings of the case to date, discuss the implications of those findings, and form the next phase of the study. Thus, it developed organically from the input of focus group participants, discussion groups, and utility stakeholders.

### 2.1. Research Methods Phase 1: Focus Groups

The research team conducted focus groups to document their thoughts, feelings, and perceptions about the safety of their tap water as well as their understanding of water quality contaminants. Focus groups were chosen for this project as the exploratory or hypothesis generation phase [30]. Their transcripts provide well-formulated accounts of the topics under study and observation of collective sense-making in action. They also provide an understanding of participants’ concepts and concerns [31].

To prepare for the focus groups the research team attended a professional workshop to learn how to conduct focus groups successfully. The training laid the foundation for how the focus group sessions were structured, organized, conducted, and evaluated. Ultimately, the research team wanted to answer the following questions:

1. What is your confidence level in the safety and quality of tap water?
2. On an average day, do you drink tap water, bottled water, or water after additional treatment? (Brita filters, refrigerator, and other filters)
3. If only unfiltered tap water is available, will you drink it?
4. Can you describe or define what a contaminant is to you?
5. If there was a contaminant in your drinking water, would you feel at risk?
6. What communication sources are you most likely to hear about a new contaminant?
7. Would the way you hear about a new contaminant affect how severe you believe it is?
8. What questions would you ask if a new contaminant were to be discovered in the water?

The purpose of the focus groups was to provide qualitative information about effectively identifying issues surrounding risk communication as the basis to improve communication between the community and the utility. Therefore, the focus groups were deemed to be evaluation tools and not to be Human Subjects Research by the University of Arizona’s Institutional Review Board.

A total of eight focus groups were conducted using four age groups, with two focus groups being conducted per age group. Ages ranged from 16 years old to over 55 years old and were divided into four groups: 16–18 years old, 18–29 years old, 30–54 years old, and 55 years and older. The age ranges were chosen to best represent the Tucson population and to determine if there were differences in the understanding and perceptions of water quality and contaminants. Each participant received a $25 gift card, and a total of 67 people participated in the focus groups.

Participants were recruited from various areas in Tucson to represent various neighborhoods. The 16–18-year-old age group was recruited by high school teachers and conducted as a part of a class. Other age groups were recruited through community organizations as well as social media and flyers. Recruitment presentations were also given in large classes on the University of Arizona campus.

Focus groups were recorded and transcribed verbatim by a transcription service. After completion of the focus group sessions, the transcript was coded. These codes were then analyzed to identify and evaluate common themes summarizing participants’ results. Codes were then discussed as a team and tabulated to identify common themes that summarized participants’ responses.

#### 2.1.1. Analysis Coding of Focus Group Transcripts

The focus groups were conducted over two semesters. They were recorded and transcribed verbatim. Four staff members and four interns conducted the focus groups and analyzed the transcripts based on a grounded theory method used in previous research [2]. The team also kept large post-it notes to record the responses of focus group participants.

Transcripts were analyzed using a grounded theory approach [32]. After each, the transcripts were reviewed by one person who conducted the focus group who and evaluated the responses into codes. Transcripts were then exchanged with another person on the team to recode them and allow for agreements and discussion. Where there was no agreement, the section of the transcript was further reviewed by the entire team. Comments were labeled if they were not relevant to the focus group questions. After exchanging transcripts, the entire team met to make suggestions for existing or new codes or to confirm agreement with existing codes. New codes or sub-codes were added to an overall list. Emergent codes were discussed in depth among the team to further develop the coding framework and to guide avenues of exploration in subsequent focus groups.

The codes were grouped to form categories [32]. This process of “researcher triangulation,” in which more than one researcher analyzes the same data, produces rigorous data as their different perspectives serve to confirm the developing themes [33,34]. A total of eight common codes were identified from these focus groups, which were further defined into twenty-five Sub-Codes and fifteen 2nd Level Sub-Codes. Not all Codes had 2nd Level Sub-Codes.

#### 2.1.2. Focus Group Codes

Table 1 describes the codes that were identified through the content analysis process. The first column (Code Name) of the table describes the general topics that participants discussed, such as the reasoning behind their perceptions of water quality and why they choose specific drinking water sources or the types of information sources they typically use and trust. The second column (Sub-Code Name) further clarifies what the code category is referring to, and the third column (2nd level Sub-Code) further describes the specifics of participants’ answers. The last column provides definitions for each of the codes used. There are some code categories that do not use a 2nd level sub-code.

### 2.2. Research Methods Phase 2: Checklist Development

Results from all eight (8) focus groups were used to develop a checklist for materials development. To create the checklist for message and materials development, four members of the research team reviewed the codes to identify the essential features. This was conducted in group sessions and then shared with the authors of this article. Table 2 identifies the specific characteristics needed for materials development.

This checklist was used in a pilot test to develop messaging and accompanying pilot materials using a question the research team often hears, “Why is the water cloudy? It looks dirty.” The answer was that the water was cloudy or turbid because of air bubbles in the water that resulted from changes in the temperature of the water in the system. The materials all bore the same message but were in several formats, such as radio spots, video shorts, flyers, infographics, and a PowerPoint presentation.

These materials were evaluated by public health college students. The evaluation method was utilized in three classroom discussion activities about the “cloudy water” materials using the questions in Table 3. Student responses were recorded during the evaluation sessions and discussed with the study team. The findings of those evaluation sessions were summarized for the next phase of the study, protocol development.

### 2.3. Research Methods Phase 3: Protocol Development

From an assessment of the checklist and suggestions from the pilot project concerning “cloudy water,” the protocol was developed. Important steps included:

- Consider the audience.
- Develop the messages about the contaminant.
  ○ Risk information
  ○ Information about safety from the contaminant
- Develop materials specific to the audience or audiences.
- Evaluate the materials with members of the intended audience.
- Implement materials in a timely fashion.
- Recommunicate with audiences in long term contamination.
- Evaluate the process.

It became clear from conclusions drawn in monthly meetings of the team from all the previous phases that the audience needs to be engaged to get their attention. Without such engagement and attention, they are unlikely to understand the message or materials. This point led the team to prioritize audience characteristics as the very first step in the protocol. Several indicators may influence the way to communicate with the audience, such as their perception of risk, their trust of the information provider, their race and ethnicity, health literacy levels, and age, among other demographics. Within the protocol, we stress that the target audience should influence the nature of the educational materials, the method of communication, who should provide the communication, and the language to be used. All other components of the protocol, messages, both risk and safety, timing of the communication, types of materials, and methods of evaluation, came after audience characterization because, without that essential piece, everything else following after will be incomplete.

### 2.4. Research Method Phase 4: Training Module

An online training curriculum module was created in the Google Classroom platform, entitled Risk and Safety Classroom https://classroom.google.com/u/0/h (accessed on 18 April 2022). The goal was to teach water utility communicators, public health, and public information officers how to use the Risk and Safety Communication Model effectively to create messages and materials that are understandable to the intended audience. Important to include were teaching about developing understandable messages to communicate risk and safety and training about techniques to use in creating materials. The learning objectives are for students to be able to discuss:

1. The basics of risk communication
2. The relationship between risk perspectives and communication
3. How to understand the intended audience of messages and materials
4. Use of the Health Literacy Data Map [11]
5. The types of materials that will be most suited to each intended audience
6. The reasons for the communication, both immediate and long-term
7. The five basic questions to answer in the messages and materials
8. How a person can protect themselves, their family, and their community
9. How to evaluate the materials that are developed with the intended audience

Virtual presentations and learning materials were created to address each objective. In some cases, Ted Talks, as well as news and peer-reviewed articles, were included in the learning materials. Quizzes were created to assure students understand the lessons, and a final assignment to develop materials concerning a hazard in the local community. Feedback is available on the quizzes and final assignments if the online student desires.

The module was evaluated with a class of master’s level public health students. The online learning materials were shared with the students. The students completed the final assignment by addressing human health from environmental exposure to treated reclaimed wastewater. Lindsey evaluated the assignments using a rubric shared with the students. Evaluations were determined based on the descriptions of (1) the reason for communication, (2) clear identification of the audience, (3) providing answers to basic questions of how it is safe, what is the risk, where is the risk, identify misconceptions about the risk, and (4) providing information about how the audience can protect themselves.

## 3. Results

### 3.1. Results Phase 1: Focus Group Results

The responses were compiled from eight (8) focus groups, two focus groups per four age groups (16–18, 18–29, 30–54, 55+), considered to be representative of a cross-section of the community. Analyzing the transcripts of the focus groups, the research team developed an understanding of the informational needs of the public regarding water quality.

The results from the analysis of questions about the fictitious contaminant and the participants’ responses to questions about tap water are presented below.

#### 3.1.1. Summary of Participants Response to Fictitious Contaminant Exercise

Coding the focus group transcript allowed the research team to be able to quantify and make sense of the qualitative data participants provided. During the focus group, participants were introduced to a fictitious contaminant and were asked about the types of questions they would have about that contaminant. Table 4 shows the types of questions participants asked about the fictitious contaminant presented in the exercise.

This information was essential in developing the Risk and Safety Communication model and identifying the types of information that would need to be included in communication materials and campaigns.

Next, participants were asked how they would be most likely to first hear about the contaminant. Participants indicated that they are most likely to hear about a new contaminant in the water through TV, friends, family, the news, online/social media, scientists, and University and government sources.

Participants were then asked about how the way they first learned about the contaminant would affect how severe, risky, or safe they perceived the contaminant to be. Participants indicated that if the information came from TV, News, radio, and governmental sources, the information is considered more severe compared to social media articles or handouts.

In terms of the types of sources that participants trust, participants seemed to trust universities and medical professionals while having a distrust for government entities, including the water utility company. Some groups reported the University of Arizona and the News as the most trusted, with the utility as the least trusted sources.

#### 3.1.2. Summary of Participants Responses to Question about Tap Water

**Figure 1:**
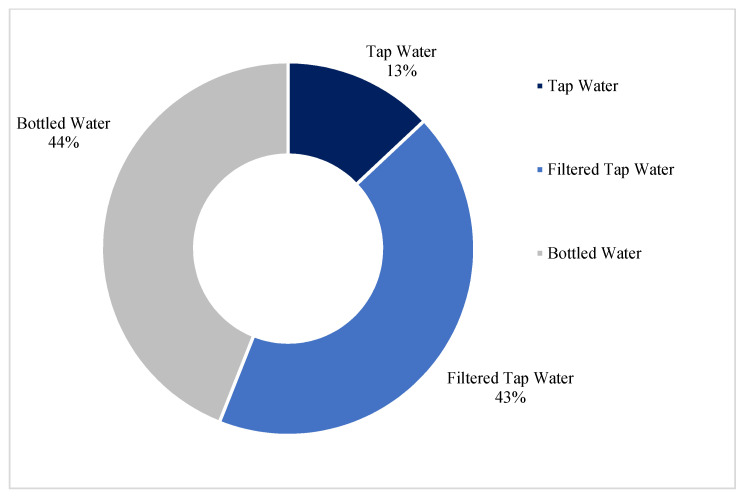
Main Sources of Drinking Water.

**Figure 2:**
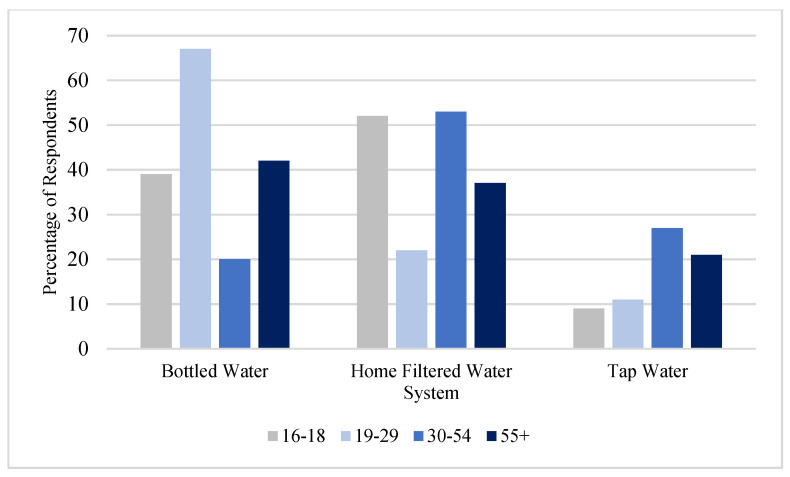
Tap Water Preference by Age.

Figure 1 shows where the participants obtain their drinking water, and Figure 2 indicates the preferred method of consuming drinking water displayed by age group. Results of the drinking water question indicate the participants primarily identify bottled water or filtered tap water as their main sources of drinking water, and although the results varied by age group, this same pattern was observed where the participants’ preference was to consume bottled water and filtered tap water versus consuming tap water.

**Figure 3:**
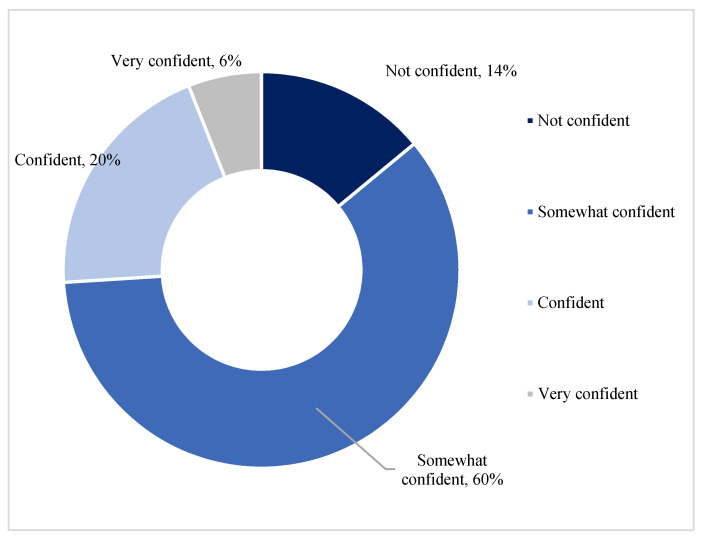
Confidence in the Quality of Tap Water.

**Figure 4:**
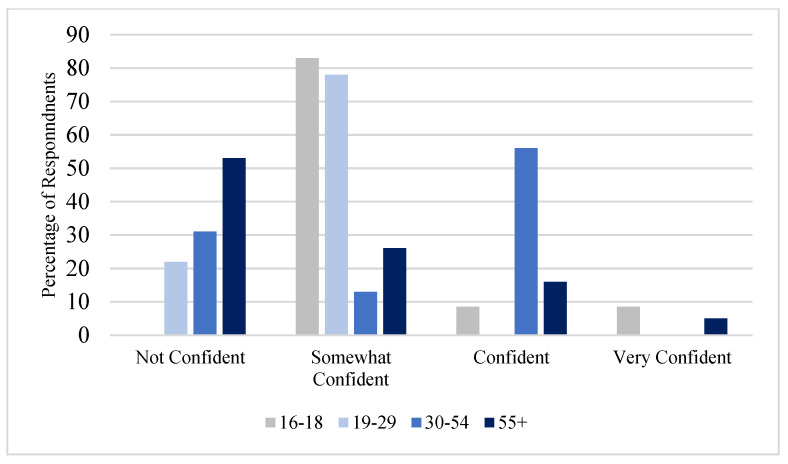
Confidence in Tap Water by Age.

Figure 3 indicates the participant’s confidence levels in the quality of tap water, and Figure 4 indicates the confidence levels by age group. The measure of confidence in the quality of tap water ranged from very confident to somewhat confident, with 14% indicating not being confident in the quality of tap water and 6% indicating that they were very confident. The lower confidence in the quality of tap water was largely among older participants, with younger participants being more confident in the quality of tap water.

### 3.2. Results Phase 2: Checklist

Important findings that developed into the checklist were (1) identifying the characteristics and communication needs of general and targeted audiences, (2) using non-scientific language or plain language, (3) providing basic information needed for clear communication, and (4) evaluating the messages and materials before disseminating to targeted audiences.

Some additional questions and suggestions came from the pilot project discussions concerning cloudy water, which enhanced the definitive version of messages and materials. The students suggested the following to make sure that the information was useful:

- Determine the level of environmental literacy levels of the intended audience.
- Determine what the audience knows before they are exposed to the materials.
- Deliver the message as a story rather than as a lecture.
- More talking with less PowerPoint.
- Use a conversational tone.
- Communicator should have applicable knowledge as well as the personality and presentation skills to engage the audience.
- Make an emotional tie to all information to address their perceived risk.
- Use anecdotes, stories, narratives, or examples to make data come alive.
- Use risk comparisons; they must consider the distinctions the public considers important.

These classroom activities resulted in the Nine Principles of Risk and Safety Communication (Table 5). These components ensure that the needs of the target audience are the focus during the development/creation of materials and messaging.

#### 3.2.1. Nine Principles of Risk and Safety Communication

Nine principles of risk and safety communication were established in this study which includes the audience, the content, and evaluation. Table 5 identifies (1) understanding the audience for the communication, (2) describing the content from a risk standpoint and a safety standpoint, how the audience can protect themselves, their families, and their community, and (3) how to keep the messages and the materials current and accurate.

Specifically recognizing audience characteristics is fundamental to effective communication, especially when presenting information about environmental concerns, which is often emotionally charged for recipients of the information. Therefore, building trust is essential. This means communicating regularly with the audience in simple terms and with methods that are acceptable to them when there is not an emergency as well as when there are environmental issues in the community. The communicator must understand the essential demographics of the audience they want to reach, such as ages, socioeconomic level, educational background, culture, language, and history with environmental concerns. In a community that has experienced environmental issues in the past, it is especially important to have excellent communication practices.

Content development comes after the audience is well characterized and may take different forms for different ages, cultures, or language groups. There are six principles concerning content which include taking into consideration how the audience perceives the risk and using varied materials for different ages, cultures, and language groups. Important for all audiences to know is the reason for the communication, that the messages answer the five basic questions clearly in plain language and that the images match the messages. Plain language is “Reader-centered, in active voice, not passive, short sentences and paragraphs, common, everyday words and easy-to-follow design features (lists, headers, tables)” [3].

For trust to be maintained, materials and messages need to meet the information needs of the audiences over time. Evaluation of the materials and messages in focus groups, surveys, and one-on-one interaction with individuals at public events is imperative.

#### 3.2.2. Results Phase 3: Protocol Development

It is the responsibility of the presenters/communicators to know the perceived risk of the audience and to identify their understanding levels to make the messages useful. Different age groups and cultural perspectives require different media and language for the materials to be developed. It is essential to understand the perceived risk on the part of the intended audience. Messages need to be changed into plain language so that it is understandable to the specific audience.

Using the Health Literacy Map [8] as a resource to understand the comprehension level of the audience can be useful in creating understandable messages. Data are available at the census block level, and there are clear descriptions about understanding that data.

In addition, presenters, communicators, and creators of messages also need to determine the perceived risk of the audience. This is best achieved through direct communication with the audience. Community meetings can be effective in gathering information about how community members perceive the problem. This step is important to make the final messages useful. There should be materials that express the simplest message and materials with more detail. Many people only want to know the basic information, while others want to know the details. The Risk and Safety Communication Model protocol guides the presenter through the process for all steps to be completed, and the materials and messages are accurate and understandable to the intended audience.

**Figure 5:**
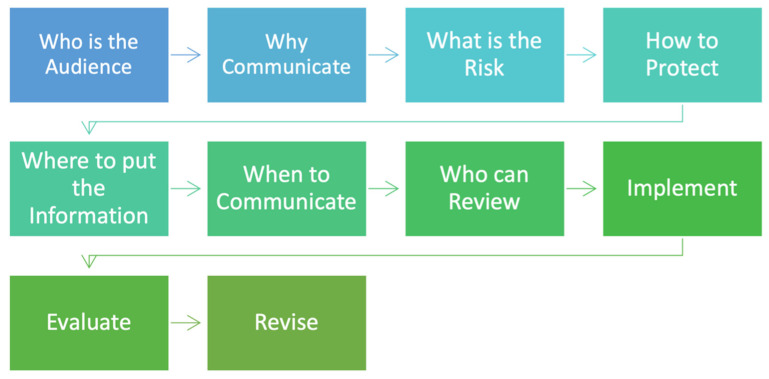
Risk and Safety Communications Model.

The Risk and Safety Communication (Figure 5) begins with learning about the target audience characteristics and understanding how to use the inputs from the protocol to develop a tailored communication model for the target audience. The inputs include general age, gender, culture, education level, health literacy levels, language preference, and socioeconomic levels.

As a utility communicator, public health professional, or public information officer, you need to determine why you are communicating the information. Is it an emergency, are there new laws or rules, and is there increased public or media interest in the issue? In other words, why do we care?

Next, form the risk messages to communicate, taking into consideration the community’s history of trauma and distrust concerning water quality [35,36]. Learn to communicate in plain language by finding suitable wording to explain complicated health, environmental, and scientific concepts. Address any misconceptions that need to be overcome. Including protection/safety messages to explain health concerns [37] and how the public can protect themselves, their families, and their community by answering these basic questions, (1) Is it safe? What is it? (2) Where is it? (3) What are the utility and public health services doing about the issue? (4) What can the individual do?

After the messages have been written, including addressing how the public is perceiving the risks, begin to consider the materials. Identify the appropriate method of communication with the intended audience; internet, press releases, print media, television, radio, public events, social media, and flyers. Think about relationships you have with other credible sources to gain third-party support for messages.

The immediacy of the risk will determine when to communicate. The principle is to be transparent in the communication. Therefore, as soon as the messages can be created and evaluated, they should be disseminated. With long-term problems that take several years to remediate, it is important to update messages and materials and periodically share them with the audience.

Create messages and materials and evaluate them with the intended audience for clarity and comprehension and peer review to assure accuracy and check for spelling and grammar. Answer the questions in Table 6.

Depending on whom the messages are intended to reach, the implementation may take many forms since one flyer, PSA, or internet posting is not likely to reach all the people in the intended audience. Make sure that the implementation plan includes a variety of information methods.

Evaluate the messages for continuity, clarity, and consistency. Re-write messages until they are in plain language by expressing one idea at a time, make the message personal for the audience, find a way to be positive, use meaningful visuals, and explain the reason for the problem and how the remediation is happening. Tell stories about people and suggest actions. Continue creating and evaluating messages and materials as needed to reach a variety of audiences to be engaged.

### 3.3. Results Phase 4: Training Module

The training module includes introductory information on how to identify the target audience and provides a step-by-step process to create tailored messages and materials at the virtual Risk and Safety Classroom https://classroom.google.com/u/0/h (accessed on 18 April 2022). All students in the evaluation of the training module were able to create meaningful materials that demonstrated comprehension of the Risk and Safety Communication Model. The content of the module includes the following:

- Introduction to the broad strokes of Risk and Safety communication, its importance, and how it is implemented to a larger community audience.
- Discussion of the importance of an understanding of risk perception and plain language for effective communication
- Description of the Risk and Safety Model for developing messages and materials as well as the various media for different audiences
- Using the evaluation checklist to ensure all components of the protocol are included in the creation of messages and materials.
- An assignment to describe the community audience and any hazards that are present to create a fact sheet, presentation, or social media regarding the hazard and how the community can protect themselves, their family members, or their community. A list of other materials to include should be created but not developed.
- To access the site, potential students are asked to contact Ben Richmond at richmond@pharmacy.arizona.edu.

## 4. Discussion

The Risk and Safety Communication Model was developed from the perspective that customary/traditional risk communications guidelines/methods often do not reach the intended audience. Traditional methods may not consistently communicate the actions the affected community should take to keep themselves, their families, and their communities safe from exposure to environmental hazards. Miscommunication leads to mistrust and, ultimately, noncompliance with risk communication messages. Often to be comprehensive in risk communication, the safety messages are missing or not evident. A recent Google Scholar search for “risk communication safety messages” revealed two international articles and one cited [35,36] that addressed safety messages in graphical materials.

The developed model is based on theoretical foundations in environmental health literacy, risk perception, and plain language. Additionally, it serves as a guiding protocol to create, evaluate, and incorporate audience criteria as inputs into creating tailored messages and materials.

The Risk and Safety Communications Model provides the nine principles of risk and safety communication as a foundation and ten (10) essential inputs (Figure 1) used to build the model. Unlike traditional/customary guidelines, the model is flexible in that the creator/communicator can select the applicable inputs to produce an effective and easy-to-understand message about a sensitive topic affecting the community. The model did not deviate from core standards of public participation and engagement [25]; instead, the model identifies critical inputs that focus on audience characteristics and the environmental health literacy of the audience that is the basis for developing focused message content using plain/simple language and delivery methods.

### 4.1. Lessons Learned

The results of the study indicate that a customary/traditional approach to communicating about risk and safety may neither include collecting essential information about the audience nor how to use that information to create a dialogue with the member of the affected community. In addition, traditional/customary risk communication guidelines state what to include in risk communication but do not provide how to develop an outreach and education plan.

The focus of this project was to develop a model which allows for audience differences in order to create focused messages and materials concerning tap water quality and information to address short-term and long-term contamination issues and focus on safety messages as well as information about risks. Testing of the model to address other environmental exposures was not undertaken, being seen as outside the scope of this project. This is one limitation of the study. Another limitation is that only one community participated in the study. Thus, the applicability of the model to environmental exposures in other communities and about other sensitive topics is limited and should be expanded in future research.

The model may be applied to effectively communicate with the community because it focuses on the intended audience, being transparent about the facts, using simple language, and communicating safety as well as risk.

### 4.2. Limitations and Future Research Directions

Limitations include having small numbers of participants, only conducting the study in Tucson, and a lack of follow-up with a survey. Focus groups have general limitations of the possibility of a dominant voice, bias from moderator influence, and the collective voice that develops may be already held views, or individuals may be influenced by others in the group [38]. In addition, the information developed by a focus group process is only reflective of each group at the specific time the focus group was held.

However,

“*the strength of focus groups lies not in quantitative analysis or in making statistically probable generalizations but in the fact that focus groups can show some evaluations, approaches, and mechanisms that exist in the target population, and they can provide characterization of the phenomena studied. Focus groups can help in building theories*”.

[37]

The next step in the study is continuing the use and development of the model and having communicators access the online training module at the virtual Risk and Safety Classroom https://classroom.google.com/u/0/h (accessed on 18 April 2022). Additional use of the model will provide the research team with essential information on the performance of the model and ways to improve and refine the inputs and outputs.

### 4.3. Novelty of This Project

The significance of this project was establishing the importance of three elements to risk communication. The authors have been at the forefront of studying environmental health literacy as an attribute of effective risk communication. Others, some within the environmental health sciences community engagement cores, have begun to study how EHL can improve risk communication [20,39,40,41]. An understanding of the health literacy of a community or census tract can be a substitute for understanding the EHL of the public [42].

In this study, it was clear that risk communicators needed to pay attention to the characteristics of their audience and to be outwardly focused rather than focusing on the details of the information about the risk, both scientific and engineering. While others have pointed out the need to attend to the audience [43,44,45], not many have studied this aspect of risk communication.

Providing information about actions the public can take to keep themselves, their families, and their communities safe is also under-studied and under-emphasized in descriptions of risk communication. Recent literature includes the public’s perception of risk [46] and improving the public’s confidence in their tap water [47,48] rather than improving the communication about water.

## 5. Summary and Conclusions

One participating intern said, “As an undergraduate, I spoke about my studies in a way that I understood, but not in a way that my peers understood. When I was asked to lead the development of the risk communication protocol, I began to realize how imperative it is to tailor your communication to your audience.”

### 5.1. Recommendations

This article proposes some new best practices for engaging with stakeholders and audiences about environmental health conditions. Do not assume that the messages are going to be trusted or understood. Instead, learn about and consider the history of past trauma and trust levels between the public and the water utility. Learn the characteristics of the community, specifically the HL level, to be able to consider the EHL of the community when forming messages and materials. Finally, be in constant communication that includes safety information and a citizen panel that can review messages and materials before they are finalized and shared with the community. Evaluate the effectiveness of the information dissemination and revise as needed.

Developing a profile of the community in advance will aid when an emergency arises, and communication is needed quickly. It is critical for utility communicators and public health professionals to develop an understanding of the educational and literacy levels and cultural-language backgrounds of the people they serve. Doing this process with a community advisory board can build credibility, trust, and transparency. That same understanding will aid in discussing an ongoing issue or updating the community about urgent actions they may need to take in response to a concern affecting the community.

Including the risk perception of community members of all ages, educational levels, and socioeconomic status is likely to provide a critical input that will help address their concerns and worries. It is essential for communicators to understand the perceptions and misconceptions the community have about risk and safety issues and events to address and dispel those perceptions and misconceptions. This finding cannot be ignored or downplayed, as doing so will lead to eroded trust, which is essential to have established with the audience for effective risk and safety communication.

Another essential skill is to use plain language to create focused messages and materials that use non-scientific terms but do not “dumb down” the message. Using plain simple language will facilitate sharing scientific concepts that address risk and ways the affected community can protect themselves, their families, and the community, will engender trust and advocates for the utility.

### 5.2. Conclusions

The Risk and Safety Communication Model was developed to demonstrate how risk and safety communication can be aligned to improve the interaction with all audiences, regardless of the level of education, to effectively communicate information on how the audience can remain safe from environmental exposures. It also provides resources and recommendations on how to apply the model when there is a need to communicate to diverse audiences about risk and safety issues.

An unanticipated outcome was the development of an online training module that includes introductory information about the model, techniques on how to identify the target audience, and provides a step-by-step process on how to create tailored messages and materials. The online training module is available to utility communicators, public information officers, and public health professionals, at the virtual Risk and Safety Classroom https://classroom.google.com/u/0/h (accessed on 18 April 2022). The model provides a standardized approach to initiate a dialogue with a community regarding risk and safety issues such as water quality that can lead to creating communication profiles of the diverse audiences within a community.

The findings of this research confirmed that risk communication needs to include and align safety information and take into consideration the public’s feelings, concerns, and perceptions about risk and safety to develop focused messages and materials that build trust and confidence. Important outcomes of this research are expanding the focus of risk communication to a genuine understanding of the public’s perception of risk and historical trauma concerning public drinking water contamination. This compassion leads to the importance of knowing the characteristics of the public, including their health literacy levels, as surrogates for their environmental health literacy levels. This reinforces the importance of communicating how people can keep themselves, their families, and their communities safe while providing risk information in plain language rather than jargon and scientific terminology.

## Figures and Tables

**Table 1:** List of Codes.

Code Name	Sub-Code Name	2nd Level Sub-Code	Description
Reasoning	Reasoning-Safe	Reasoning-safe-explanation: clear, boiling water, informer, have not become sick	A participant’s stance and explanation about the safety of their water.
	Reasoning-Avoided	Reasoning-avoided-explanation: taste, feels funny, not clear	A participant’s stance and explanation about the concerns they identify in their water.
	Reasoning-Bottled Water	Reasoning-Bottled Water: taste, convenient, available	A participant’s choice to drink bottled water, and factors leading to that decision.
	Reasoning-Tap Water	Reasoning-Tap Water: bottles, expensive	A participant’s choice to drink tap water due to prohibitive cost of other forms.
		Reasoning-Tap Water: environmentally better	A participant’s choice to drink tap water due to environmental factors.
		Reasoning-Tap Water: convenient, mixes to make other drinks	A participant’s choice to drink tap water due to convenience and easy use with other products.
		Reasoning-Tap Water: forced	Participants are only willing to drink tap water when there are no other options.
	Reasoning-Filtered Water	None	A participant’s choice to drink filtered water.
	Reasoning-Confidence level	Reasoning-Confidence Level: Level of confidence specified	A participant’s confidence or lack of confidence in their drinking water.
	Reasoning-Last Resort	None	A participant’s choice to only drink a form of water in a dire situation.
	Reasoning-Feeling of risk	Reasoning-Feeling of risk: Level of risk specified	A participant’s feelings of risks around drinking water.
Treatment	Treatment-specific (ex: Brita)	Brita, fridge, filter	A participant’s actions to treat their water before consumption.
Consumption Pattern	Consumption Pattern-Regularly	Consumption Pattern-Regularly: Type of Water	How often and/or what type of water they consume.
	Consumption Pattern-After Treatment	None	Alternative consumption pattern based on a treatment in the water.
	Consumption Pattern-Changing	None	A change in behavior around drinking water.
Demographics	Demographics-Birthplace/origin	None	A participant’s birthplace/place of origin.
	Demographics-Residence	Demographics-Residence: Time in Tucson	A participant’s area of residence, and how long they have resided in an area.
	Demographics-Education	None	The educational background of participants.
Definition	Definitions-Contaminated Water	Definitions-Contaminated Water-Example	A participant’s definition or example of contaminated water.
Information Source	Information Source-Influence	None	A participant’s beliefs surrounding the influence of an information source.
	Information Source-Trusted	Information Source-Trusted-Specific Media: news, social media, Twitter, water company/utility	A participant’s trust in an information source.
		Information Source-Trusted-Level of Trust/Credibility	A participant’s belief in the credibility and/or level of trust in a source.
	Information Source-Type of Source Specified	None	A specific type of source that is sought out for information.
	Information Source-Severity	None	A participant’s belief that a type of information source means the information is more severe.
Information Seeking	Information Seeking-Questions	None	A participant is asking questions about information that is wanted.
	Information Seeking-Concerns	None	A participant has concerns about some information and would more information to address them.
	Information Seeking-Answers	None	Provide answers to participant questions or concerns.
Preexisting Knowledge	Preexisting Knowledge-Contaminants	None	Prior knowledge about contaminants that participants brought in.
	Preexisting Knowledge-Example of World Events	None	Knowledge about current world events that serve as examples of information in the focus group.

**Table 2:** Checklist Elements for Materials Development.

Information needed for effective message and materials development:
Information about why to communicate, emergency or ongoing Information about audience perception about the water concern Location (zip code) affected Age of the intended audience Demographics; culture, language, education, preferred language Does it answer the following essential questions? What is it? Where is it? Is it safe? What are you doing about it? What can I do about it? Is the information credible? Is the information clear? Is the information consistent and persistent? Does it reach vulnerable populations?

**Table 3:** Discussion Questions for Materials Evaluation.

How does it address the following? ○ Uncertainties ○ Strengths ○ Weaknesses How does it meet the needs of different audiences? How does it respect the needs of different audiences? How is it culturally competent? How does it acknowledge cultural views and norms? Is it sensitive to those views and norms? How does this make the target audience feel? ○ More informed ○ That their voice is heard ○ Respected Does this reach the target audience in the most effective way? Why or why not

**Table 4:** Participants’ questions about the fictitious contaminant.

Who is affected? How do you come into contact with the contaminant? What are the symptoms and severity of illness? Is it treatable? Could it be removed from the water? Do daily activities need to be adjusted to avoid the water? Where is the contaminant coming from? How long have we known there is a contaminant present? What damage will the contaminant leave in the environment?

**Table 5:** Nine Principles of Risk and Safety Communication.

Process—Understanding the Audience for Communication Build the trust needed for excellent communication. Prepare for a specific audience. Content—Describing the content from both a risk and a safety standpoint 3. Actual and perceived risk. 4. Accommodate for differences among people. 5. Address the reason for communication. 6. Suggest actions people can take in case of risk 7. Answer basic questions with clarity, continuity, and consistency. 8. Images match the content message. Testing—To keep the messages and the materials current and accurate 9. Evaluate and improve messages and materials.

**Table 6:** Evaluation Questions for Risk and Safety Communication Materials.

Did the messages and materials reach the desired audience and develop trust for the provider?
Did the messages and materials answer the audience’s questions? ○ Is it safe? ○ What is it? ○ Where is it? ○ What are the utility and public health doing about the issue? ○ What can the individual do?
Did the participants: ○ understand the risk and safety information, ○ want more information, ○ feel respected by the provider, ○ plan to use the information, or ○ have questions that were not anticipated?

## Data Availability

The data presented in this study are available on request from the corresponding author. The data are not publicly available due to funding restrictions as specific to and proprietary to Tucson Water.
